# Characterization of the complete chloroplast genome of *Bromus inermis* Leyss

**DOI:** 10.1080/23802359.2021.1972864

**Published:** 2021-09-20

**Authors:** Keyan He, Jiahao Wang, Yongchao Zhang, Yan Qin, Hao Wu, Xiaoxing Wei

**Affiliations:** a Qinghai Academy of Animal Husbandry and Veterinary Sciences, Qinghai University, Xining, Qinghai, China; b Northwest Institute of Plateau Biology, Chinese Academy of Sciences, Xining, Qinghai, China

**Keywords:** *Bromus Inermis* Leyss, chloroplast genome, phylogenetic relationship

## Abstract

*Bromus Inermis* Leyss 1761 is a perennial herb with large yield and famous for its high nutritional value and long utilization season. The study of *B. inermis* chloroplast (cp) genome provides an important basis for the study of chloroplast genetic engineering and system evolution. Its cp genome was 137,153 bp in length, containing a pair of inverted repeated (IR) regions (21,699 bp), separated by a large single copy region (LSC) of 81,137 bp, and a small single copy (SSC) region of 12,618 bp. Moreover, a total of 129 functional genes were annotated, including 83 mRNA, 38 tRNA genes, and 8 rRNA genes. The phylogenetic relationships of 15 species indicated that *B. inermis* was closely related to *Bromus biebersteinii*. This study might contribute to provide a theoretical basis for species identification and biological research.

*Bromus inermis* Leyss belongs to the perennial poaceae plants. Its life span can be more than 30 years, and it is an important cultivated grass in arid and cold regions (Shi et al. 2007). Its wild species are widely distributed in temperate regions of Eurasia, and are widely distributed in Gansu, Qinghai, Tibet and other provinces of China (Gong et al. 2019). DNA barcodes derived from cp genome will be useful for identifying varieties and resources; this concept is also valuable in the identification of the origin of cultivated crops and their close relatives to enhance breeding or transfer of useful traits (Henry et al. 2016). However, most of the studies on *B. inermis* were on the identification of genetic diversity of its germplasm resources, cultivation management and response to adversity, etc., while few studies were on the determination of its genetic relationship by the genome sequencing. Therefore, this study determined its closely related plants by measuring the genome, providing certain reference for the selection and breeding of excellent varieties. In this study, the cp genome sequencing of *B. inermis* (GenBank accession number: MW861351) was done by Illumina NovaSeq platform, and the genome sequence structure was analyzed, which would provide a basis for the judgment of the relationship of related species and genera.

The fresh young leaves of *B. inermis* were collected in Xihai Town, Haibei Prefecture, Qinghai Province, China (E100°52.848′, N36°59.36′). The voucher specimen was kept in Herbarium of Low Temperature Bank of Germplasm Resources of Qinghai Academy of Animal Husbandry and Veterinary Sciences (contact person and email: Xiaoxing Wei, wuiko@163.com) under the voucher number 09-112. The total genomic DNA of *B. inermis* was extracted from the fresh leaves with a modified CTAB method (Li et al. 2018). One library was constructed using PCR amplification. The template size was 300 bp. The genome sequencing was performed with an Illumina Novaseq platform (Genepioneer Biotechnologies Inc., Nanjing, China). Finally, about 5.90 GB of clean data were generated and the SPAdes (Bankevich et al. 2012) was used to assemble the cp genome, based on the clean data. The assembled genome was annotated using CpGAVAS (Liu et al. 2012).

The complete cp genome of *B. inermis* is 137,153 bp in length, exhibiting a typical quadripartite structure including a pair of inverted repeated (IRA and IRB) regions (21,699 bp) that are separated by a large single copy (LSC) region of 81,137bp, and a small single copy (SSC) region of 12,618 bp. The GC content of the whole cp was 38.36%. A total of 129 functional genes are annotated, including 83 protein-coding genes (mRNA), 38 tRNA genes, 8 rRNA genes.

Phylogenetic analysis was completed on an alignment of concatenated nucleotide sequences of the cp genomes of *B. inermis* and other 15 species (*Pisum sativum, Medicago sativa and Medicago hybrida* as the outgroup species. Figure 1). The alignment was conducted by MAFFT version (https://mafft.cbrc.jp/alignment/software/algorithms/algorithms.html) and the Maximum Likelihood (ML) method from MEGA7.0 (Kumar et al. 2016) was employed to build a phylogenetic tree with 1000 bootstrap. The results showed that *B. inermis* had a closer relationship with *Bromus biebersteinii*.

**Figure 1.**
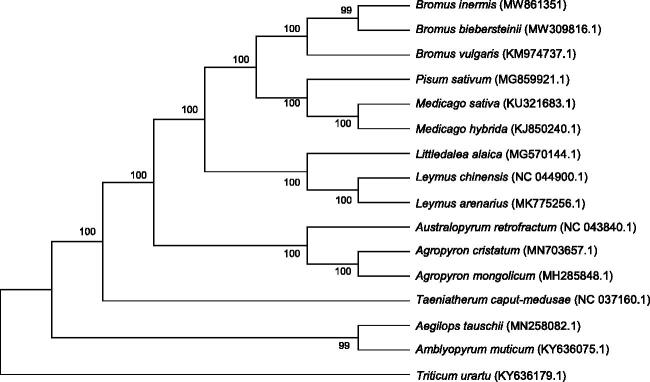
Phylogenetic relationships of 16 species based on complete chloroplast genome using the maximum likelihood methods. The bootstrap values were based on 1000 replicates and are shown next to the branches.

## Data Availability

The genome sequence data that support the findings of this study are openly available in GenBank of NCBI at (https://www.ncbi.nlm.nih.gov/) under the accession no.MW861351. The associated Bio-Project, SRA, and Bio-Sample numbers are PRJNA719012, SRR14127386, and SAMN18580319 respectively.
